# Inclusion of unaffected sibs increases power in model-free linkage analysis of a behavioral trait

**DOI:** 10.1186/1471-2156-6-S1-S22

**Published:** 2005-12-30

**Authors:** Sabine Plancoulaine, Alexandre Alcaïs, Yue Chen, Laurent Abel, France Gagnon

**Affiliations:** 1 Laboratoire de Génétique Humaine des Maladies Infectieuses, INSERM U.550, Faculté de Médecine Necker, Université de Paris René Descartes, 156, Rue de Vaugirard, 75015 Paris, France; 2 Department of Epidemiology and Community Medicine, Faculty of Medicine, University of Ottawa, 451 Smyth Road, Ottawa, Ontario, K1H 8M5, Canada

## Abstract

Study design strategies are of critical importance in the search for genes underlying complex diseases. Two important design choices in planning gene mapping studies are the analytic strategy to be used, which will have an impact on the type of data to be collected, and the choice of genetic markers. In the present paper, we used the simulated behavioral trait data provided in the Genetic Analysis Workshop14 to: 1) investigate the usefulness of incorporating unaffected sibs in model-free linkage analysis and, 2) compare linkage results of genome scans using a 7-cM microsatellite map with a 3-cM single nucleotide polymorphisms map. To achieve these aims, we used the maximum-likelihood-binomial method with two different coding approaches. We defined the unaffected sibs as those totally free of phenotypes correlated to the disease. Without prior knowledge of the answers, we were able to correctly localize 2 out of 5 loci (LOD > 3) in a sample of 200 families that included the unaffected sibs but only one locus when based on an affected-only strategy, using either microsatellite or SNPs genome scan. LOD scores were considerably higher using the analytic strategy which incorporated the unaffected sibs. In conclusion, including unaffected sibs in model-free linkage analysis of complex binary traits is helpful, at least when complete parental data are available, whereas there are no striking advantages in using single nucleotide polymorphisms over microsatellite map at marker densities used in the current study.

## Background

Affected sib-pair study designs are among the most commonly used for the study of complex genetic traits. The maximum-likelihood-binomial (MLB) approach is one such method, and unlike other sib-pair approaches, it analyzes sibships of arbitrary size as a whole. In the context of a proposed strategy to account for covariates in the MLB approach, Alcaïs and Abel have shown an increase in the power to detect a susceptibility locus when making use of the information carried by unaffected sibs [1]. The value of including the unaffected individuals *per se *in a genome-wide scan of a binary trait has not yet been investigated in the context of a sib-pair design.

To date, genome scans of complex traits have been performed using a set of 300–400 microsatellite markers (MS) evenly spaced (~10 cM) across the genome. More recently, single nucleotide polymorphisms (SNPs) have emerged as attractive alternative tools to conduct genome scans of complex traits, mainly motivated by their more rapid and highly automated genotyping as compared to MS. The merit of SNPs in the context of a genome-wide scan of complex traits is relatively undocumented. One recent study comparing MS with SNPs whole-genome scans concluded in favor of SNPs, mainly because of the more refined position of loci [2].

The purpose of our study is twofold: first, to investigate the effects of an alternative sibship design, which uses both affected and unaffected sibs, and to compare it with the frequently used affected-only design; and second, to compare genome-wide scan linkage results obtained from MS with SNPs.

## Methods

### Data

We used the simulated behavioral trait data generated in the context of the Genetic Analysis Workshop 14 (GAW14). The analyses were performed without any knowledge of the answers. For the present paper, the data from the first two replicates of the simulated population "Aipotu" were used (REP001 and REP002). In order to increase the power to detect linkage while maintaining a realistic sample size, the two replicates were combined for these analyses. The combined replicate data included 200 nuclear families. Details on the distribution of the families by number of sibs according to affection status are presented in Table 1. We used the 416 MS (7-cM scan) and 917 SNPs (3-cM scan), provided in the initial data release. It is important to note that there were no missing data. Separate analyses of the MS and the SNPs data were performed.

### Phenotype definition

The affected sibs were those with the diagnosis of the simulated behavioral trait, Kofendrerd Personality Disorder (KPD). The unaffected sibs were those not only without KPD, but also free of the 12 phenotypes associated with KPD. There was no difference in affection status according to sex or age and therefore, no covariate adjustment for these variables was done.

### Statistical analyses

Model-free two-point and multipoint genome scan linkage analyses were performed using the MLB method [3], as implemented in a modified version of GENEHUNTER[4]. The MLB method is based on the idea of binomial distributions of the number of affected sibs receiving a given allele, e.g., allele A, from an heterozygous AB parent [5]. It is a very flexible approach that can accomodate binary, quantitative, and categorical traits, and considers sibships as a whole, overcoming the multiple sibs problem. Details of the approaches used in the present paper have been described elsewhere [1,3,6-8]. Specific to this paper, we performed two types of analyses: 1) a binary trait analysis, denoted MLB-binary, based on the binomial distribution of the number of affected sibs receiving a given parental marker allele and, 2) a categorical (ordinal) trait analysis, denoted MLB-categorical, which allows the analysis of both affected and unaffected subjects (two categories). This categorical trait approach derives from the MLB extension to quantitative traits [7], which is based on the introduction of an individual latent binary variable capturing the linkage information between the observed ordinal trait and the marker. The method needs to specify the probability of the latent variable value (0/1) according to the observed phenotype (affected/unaffected). We fixed the probability to have a 1 value at 1.00 and 0.00 for affected and unaffected subjects, respectively; and to have a 0 value at 0.00 and 1.00 for affected and unaffected, respectively. This coding scheme can be understood as an analysis accounting for both extremely concordant (affected/affected or unaffected/unaffected) and extremely discordant (affected/unaffected) sibs in a sibship. The MLB binary and categorical methods are both standard likelihood-ratio statistics asymptomatically distributed as a 50%:50% mixture of χ^2 ^distributions with 0 and 1 degree of freedom; the statistic is usually expressed as a LOD score, which has exactly the same distribution as a classical model-based LOD score estimating the recombination fraction parameter [3,7]. The linkage analyses were based on the map positions provided with the GAW14 study sample and were independent of marker allele frequencies since all parents were genotyped.

## Results and discussion

**Figure 1:**
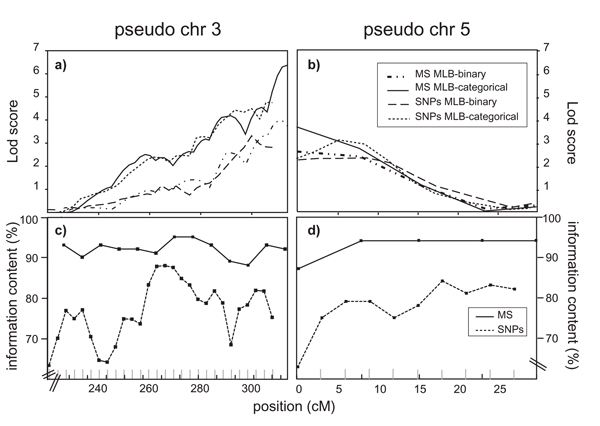
Multipoint LOD scores (a, b) and information content (%) (c, d) for the MS and the SNPs chromosome 3 (a, c) and chromosome 5 (b, d) regions for the affected-only strategy (MLB-binary) and the affected and unaffected strategy (MLB-categorical). The vertical lines on the x-axes of 1 c and 1 d are for the MS (black) and SNPs (gray) marker position. LOD and information content are provided at positions corresponding to MS and SNPs.

Figure 1 presents multipoint LOD scores and information content for the MS and the SNPs chromosome 3 and chromosome 5 regions, for the affected-only strategy and the affected and unaffected strategy.

### Affected-only strategy

The multipoint MLB-binary analysis identified one region with evidence of linkage as defined by a LOD score > 3.00, both in the MS and the SNPs scan. The peak signal is on chromosome 3 at D3S127 with a LOD score of 3.73 in the MS scan, and 3.40 at C03R0278 with the SNPs scan. Suggestive evidence of linkage is reported on chromosome 5, with a peak multipoint MLB-binary LOD score of 2.60 at D5S172 (MS) and of 2.39 at D05R0380 (SNPs). No MLB-binary LOD score above 3.00 was identified in the two-point analyses (data not shown).

### Affected and unaffected strategy

Using both affected and strictly unaffected individuals (i.e., no KPD and absence of correlated traits) leads to a marked increase in the LOD score for the signals on chromosome 3 and chromosome 5. The multipoint MLB-categorical LOD score is 6.37 at D3S217, the same marker as in the MLB-binary analysis, and 4.76 at C03R0280, a marker in the region identified in the MLB-binary analysis. The signal on chromosome 5 now provides evidence for linkage with peak MLB-categorical LOD scores of 3.67 at D5S172 (MS) and 3.14 at C05R0380 (SNPs). The increase is marked enough to be picked up as evidence for linkage even in the two-point analyses (data not shown). The peak two-point MLB-categorical LOD scores were 4.61 and 3.46 for D3S127 and D5S172, respectively. None of the two-point LOD scores for the SNPs markers were over 3.00. The higher LOD scores observed for the MS scans are likely due to the higher information content for MS vs. SNPs (Figure 1c, d). For example, the marker information content in the chromosome 3 region was on average 2.4- and 1.2-fold higher in the two-point and multipoint analyses, respectively, for the MS vs. SNPs scan; corresponding to peak LOD scores ~ 1.3- to 1.6-fold higher with the MS scan.

### Follow-up

To further investigate the two loci localized, we first aimed at identifying evidence for putative epistasis affecting the major loci identified. For this, we computed the correlation between the LOD scores at markers D3S127 and D5S172 in the 200 nuclear families. The correlation is 0.02 and non-significant (*p*-value = 0.74). Therefore, the LOD scores for these two markers are more likely independent. In a second step, we investigated putative locus heterogeneity. For this, we identified the families not contributing positively to the LOD score at D3S127, i.e., the marker providing the highest LOD score in the genome scan analysis. There were 106 such families. We then conducted two additional MS genome scans, one that included the affected-only and another that included the affected-unaffected, of the "unlinked" families. In the multipoint MLB-binary analysis, only one LOD score above 2 (LOD 2.43 at D5S203), provided some suggestive evidence for linkage. No other LOD scores, MLB-binary or -categorical, were above 2.00.

## Conclusion

In the context of sib-pair-oriented model-free linkage analysis of binary traits, the unaffected individuals drastically add to the linkage information, at least in the context of no missing data. The LOD scores were increased by up to 71% with the use of the 174 strictly unaffected individuals, i.e., 17% of the total sample ignored in the affected-only strategy. Since the cost of phenotyping is inherent to the determination of the affection status of the sibs, and because the cost of genotyping is usually low compared with the cost of phenotyping, these results are very much in favor of making use of the collected unaffected individuals. Furthermore, the strategy of including the unaffected individuals in model-free analyses of binary traits is likely to be particularly useful in the context of behavioral disorders, which suffer from limited availability of putative quantitative traits, which is another powerful strategy to tackle complex diseases. Finally, we did not observe narrower regions using 3-cM SNPs vs. 7-cM MS scan. Our results suggest that a 7-cM MS genome scan may be somewhat more powerful than a dense SNPs genome scan with maps of 3-cM, partly due to increased information content of MS.

## Addendum

Without prior knowledge of the answers, using an analytic strategy that incorporates unaffected sibs, we correctly identified two loci (LOD > 3), simulated chromosome 3 and 5 loci, with both MS and SNPs genome scans. Using a looser criterion, that is a LOD score > 1, we identified additional loci (chromosome 1 and 9 loci) with no false positives for the MS scan, but at the cost of three false positives for the SNPs scan (data not shown). The locus on chromosome 10 was missed by both MS and SNPs scans.

## Abbreviations

GAW: Genetic Analysis Workshop

KPD: Kofendrerd Personality Disorder

MLB: Maximum-likelihood binomial

MS: Microsatellites

SNP: Single-nucleotide polymorphism

## Figures and Tables

**Table 1:** Distribution of the families by number of sibs according to the affection status

Number of unaffected sibs	Number of affected sibs						Total
		
	2	3	4	5	6	7	
0	87	12	3	2	-	-	104
1	37	6	2	-	-	1	46
2	19	7	1	1	-	-	28
3	13	3	-	-	-	-	16
4	6	-	-	-	-	-	6
							
Total	162	28	6	3	-	1	200
